# Aspirin Eugenol Ester Alleviates Energy Metabolism Disorders by Reducing Oxidative Damage and Inflammation in the Livers of Broilers Under High-Stocking-Density Stress

**DOI:** 10.3390/ijms26051877

**Published:** 2025-02-21

**Authors:** Caifang Guo, Yi Zhang, Dongying Bai, Wenrui Zhen, Penghui Ma, Ziwei Wang, Xiaodie Zhao, Xiqiang Ma, Xiaolin Xie, Koichi Ito, Bingkun Zhang, Yajun Yang, Jianyong Li, Yanbo Ma

**Affiliations:** 1 Department of Animal Physiology, College of Animal Science and Technology, Henan University of Science and Technology, Luoyang 471003, China; guocaifang@stu.haust.edu.cn (C.G.); zhangyi439250@gmail.com (Y.Z.); zhenwenr@126.com (W.Z.); mapenghui@stu.haust.edu.cn (P.M.); wangziwei@stu.haust.edu.cn (Z.W.); zhaoxiaodie@stu.haust.edu.cn (X.Z.); 2 Henan International Joint Laboratory of Animal Welfare and Health Breeding, College of Animal Science and Technology, Henan University of Science and Technology, Luoyang 471023, China; 3 Innovative Research Team of Livestock Intelligent Breeding and Equipment, Science & Technology Innovation Center for Completed Set Equipment, Longmen Laboratory, Luoyang 471023, China; maxiqiang@haust.edu.cn (X.M.); xiexiaolin@haust.edu.cn (X.X.); 4 Department of Food and Physiological Models, Graduate School of Agricultural and Life Sciences, The University of Tokyo, Ibaraki 319-0206, Japan; akoito@mail.ecc.u-tokyo.ac.jp; 5 State Key Laboratory of Animal Nutrition, Department of Animal Nutrition and Feed Science, College of Animal Science and Technology, China Agricultural University, Beijing 100193, China; bingkunzhang@126.com; 6 Key Lab of New Animal Drug of Gansu Province, Key Lab of Veterinary Pharmaceutical Development of Ministry of Agriculture and Rural Affairs, Lanzhou Institute of Husbandry and Pharmaceutical Science of Chinese Academy of Agricultural Sciences, Lanzhou 730046, China; yangyajun@caas.cn (Y.Y.); lijy1971@163.com (J.L.)

**Keywords:** broilers, AEE, high stocking density, oxidative damage, inflammation, energy metabolism

## Abstract

This study aimed to evaluate the effects of aspirin eugenol ester (AEE) on growth performance, oxidative liver damage, inflammation, and liver metabolomics in broilers under high-stocking-density (HSD) stress. A total of 360 broilers were divided into four groups: normal density (ND, 14/m^2^), high density (HD, 22/m^2^), ND-AEE (ND + 0.01% AEE), and HD-AEE (HD + 0.01% AEE). HSD decreased total antioxidant capacity, increased malondialdehyde (MDA) levels, and elevated the expression of cyclooxygenase-2 (*COX-2*) and microsomal prostaglandin E synthase-1 (*mPGES-1*) mRNA, which contributed to the reduced performance of broilers. Specifically, HSD caused abnormalities in linoleic acid metabolism, leading to elevated levels of Prostaglandin E2 (PGE2) and Leukotriene B4 (LTB4) synthesis, which aggravated inflammation, increased liver lipid levels, and impaired ATP production. AEE counteracted the decline in broiler production performance induced by HSD by enhancing total antioxidant capacity, reducing MDA levels, protecting the liver from oxidative damage, and maintaining mitochondrial oxidative phosphorylation. AEE positively regulated the linoleic acid metabolism by promoting the synthesis of γ-linolenic acid and phosphatidylcholine, which reduced the synthesis of *COX-2* and *mPGES-1*. AEE alleviated the metabolic imbalance caused by HSD stress and enhanced the efficiency of mitochondrial fatty acid oxidation, which reduced excess lipid accumulation in the liver and promoted ATP production. In summary, this study provides strong support for the dietary addition of AEE to alleviate liver oxidative damage, inflammation, and energy metabolism disorders caused by HSD stress.

## 1. Introduction

In commercial broiler farming, high stocking density (HSD) is a common strategy to improve cage space utilization efficiency. While this practice promotes effective use of space, it also presents numerous challenges, including environmental deterioration, limited activity space, aggressive behavior, and increased stress, which can negatively affect the health and productive performance of the birds [1,2]. The description of high density typically focuses on two core indicators: the total weight of poultry in kg per square meter and the number of birds per square meter [3]. Considering animal welfare and growth performance, a stocking density of 16 broilers/m^2^ or 39 kg/m^2^ is considered reasonable and appropriate with little negative impact on the broilers [4,5]. Our research and studies by other researchers have shown that HSD not only reduces production efficiency but also disrupts the body’s redox balance, leading to increased production of reactive oxygen species (ROS), inflammation, and eventual oxidative liver damage [6,7,8]. ROS trigger intracellular signaling cascades in the liver that increase the expression of pro-inflammatory genes, such as tumor necrosis factor-α (*TNF-α*) and interleukin-1β (*IL-1β*), which in turn exacerbates tissue damage and oxidative stress [9,10]. With prolonged or severe oxidative stress and inflammatory stimulus, the body’s energy balance is disrupted along with lipid metabolism, causing excessive fat storage and impaired function in the liver [11]. In addition, when poultry are under stress, the hypothalamic–pituitary–adrenal (HPA) axis is activated, resulting in increased energy mobilization and altered metabolism that reduces the growth performance and feed conversion efficiency [12,13]. Existing studies have shown that HSD causes significant changes in the interconversion of pentose and glucuronic acid in broiler serum and in the pentose phosphate pathway, which is closely related to energy metabolism [14]. Despite extensive research, the specific mechanism by which HSD affects oxidative damage, inflammation, and energy metabolism disorders in broilers is not fully understood. How to effectively mitigate the damage caused by HSD remains an urgent challenge.

Aspirin eugenol ester (AEE) is an innovative compound synthesized through esterification of aspirin (acetylsalicylic acid) with eugenol. The resulting molecule combines the anti-inflammatory properties of aspirin with the antioxidant potency of eugenol while significantly alleviating the inherent limitations of both components [15]. Numerous studies have confirmed that AEE can effectively exert antioxidant and anti-inflammatory effects and improve energy metabolism [16,17,18]. AEE prevents energy metabolism imbalance and oxidative stress damage caused by hydrogen peroxide (H_2_O_2_) by increasing the activity of antioxidant enzymes, inhibiting the generation of free radicals and protecting the mitochondrial respiratory chain from damage [19]. AEE also alleviates the inflammatory response in HSD broilers by reducing the levels of cyclooxygenase-2 (*COX-2*), prostaglandin E2 (PGE2), and other inflammatory factors [20]. Under immune stress, AEE mitigates oxidative damage by enhancing antioxidant defenses, slowing oxidative phosphorylation, suppressing inflammation and reducing ROS overproduction. This improves energy production and metabolic balance by regulating phenylalanine and tyrosine metabolism and optimizing lipid and cholesterol biosynthesis [21]. To date, there has been only limited research on the effects of AEE supplementation in broilers reared under HSD. This study comprehensively analyzed production performance, antioxidant capacity, inflammation-related mRNA expression, liver histopathology, and metabolic profiling to determine the potential mitigating effects of AEE on oxidative damage, inflammation, and energy metabolism in HSD broilers. The goal was to provide scientific evidence for improving animal welfare and optimizing production performance in the poultry industry.

## 2. Results

### 2.1. Effects of AEE on Growth Performance of HSD Broilers

The effects of AEE on the growth performance of broilers are shown in Table 1. From days 1 to 14, there were no significant differences in ADG, ADFI, and FCR between groups (*p* > 0.05). From days 15 to 28, the HD group had significantly lower ADFI compared to the ND group (*p* < 0.05), with no differences in ADG and FCR (*p* > 0.05). From days 29 to 42, the HD group showed significantly reduced ADFI and ADG values and significantly increased FCR compared to the ND group (*p* < 0.05). In contrast, the HD-AEE group had significantly higher ADFI and ADG and lower FCR compared to the HD group (*p* < 0.05). The addition of AEE alone had no significant effect on the production performance of the ND-AEE group (*p* > 0.05).

### 2.2. Effects of AEE on Antioxidant Function in Liver from HSD Broilers

The effects of AEE on antioxidant indices in samples of broiler livers are shown in Table 2. The HD group had significantly reduced T-AOC and GSH-Px at 28, 35, and 42 days, with SOD activity decreasing at 28 and 35 days, and CAT activity decreasing at 35 and 42 days (*p* < 0.05). The MDA content was significantly increased at 28, 35, and 42 days (*p* < 0.05), indicating increased oxidative stress compared to the ND group. Dietary supplementation of AEE in HSD broilers improved their antioxidant status. The HD-AEE group showed a significant increase in T-AOC and SOD activity at 28 and 35 days, with GSH-Px activity increasing at 28, 35, and 42 days, and CAT activity also increasing at 35 and 42 days (*p* < 0.05). The MDA content was significantly reduced after 28 and 42 days (*p* < 0.05). No significant differences were observed between the ND-AEE and ND groups across all measured parameters (*p* > 0.05).

### 2.3. Effects of AEE on Inflammatory Gene Expression in Liver from HSD Broilers

**Figure 1:**
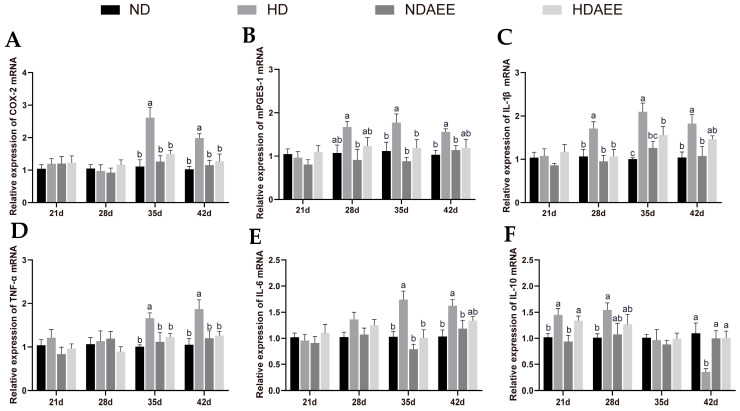
Effect of AEE on the relative expression levels of inflammatory factor mRNA in the livers of broilers across four treatment groups. ND, broilers at normal stocking density fed basal diet; HD, broilers at high stocking density fed basal diet; ND-AEE, normal-stocking-density group fed basal diet supplemented with 0.01% AEE; HD-AEE, high-stocking-density group fed basal diet supplemented with 0.01% AEE. (**A**–**F**) mRNA levels of *COX-2*, *mPGES-1*, *IL-1β*, *TNF-α*, *IL-6*, and *IL-10* at 21, 28, 35, and 42 days of age. The gene for *GAPDH* was used as a reference for normalization. Bars labeled with different letters (a, b, c) indicate significant differences across all groups (*p* < 0.05), with data presented as mean ± SEM (n = 6).

We used RT-qPCR to measure the expression levels of six inflammatory mediator genes, including *COX-2*, *mPGES-1*, *IL-1β*, *IL-6*, *TNF-α*, and *IL-10* (Figure 1). Compared with the ND group, the HD group showed a significant increase in the mRNA expression levels of *COX-2*, *mPGES-1*, *IL-6*, and *TNF-α* on days 35 and 42 (*p* < 0.05). Furthermore, *IL-1β* mRNA expression was significantly increased on days 28, 35, and 42 (*p* < 0.05). Compared with the HD group, the HD-AEE group showed a significant reduction in *COX-2* and *TNF-α* mRNA expression at 35 and 42 days (*p* < 0.05); *IL-1β* expression was significantly decreased at 28 and 35 days (*p* < 0.05); and the mRNA expression of *mPGES-1* and *IL-6* also significantly decreased after 35 days (*p* < 0.05). Compared with the ND group, the HD group showed a significant increase in *IL-10* mRNA expression at 21 and 28 days but a significant decrease at 42 days (*p* < 0.05). In contrast, the HD-AEE group significantly increased *IL-10* mRNA expression at 42 days compared to the HD group (*p* < 0.05).

### 2.4. Effects of AEE on Pathological Liver Injury in HSD Broilers

**Figure 2:**
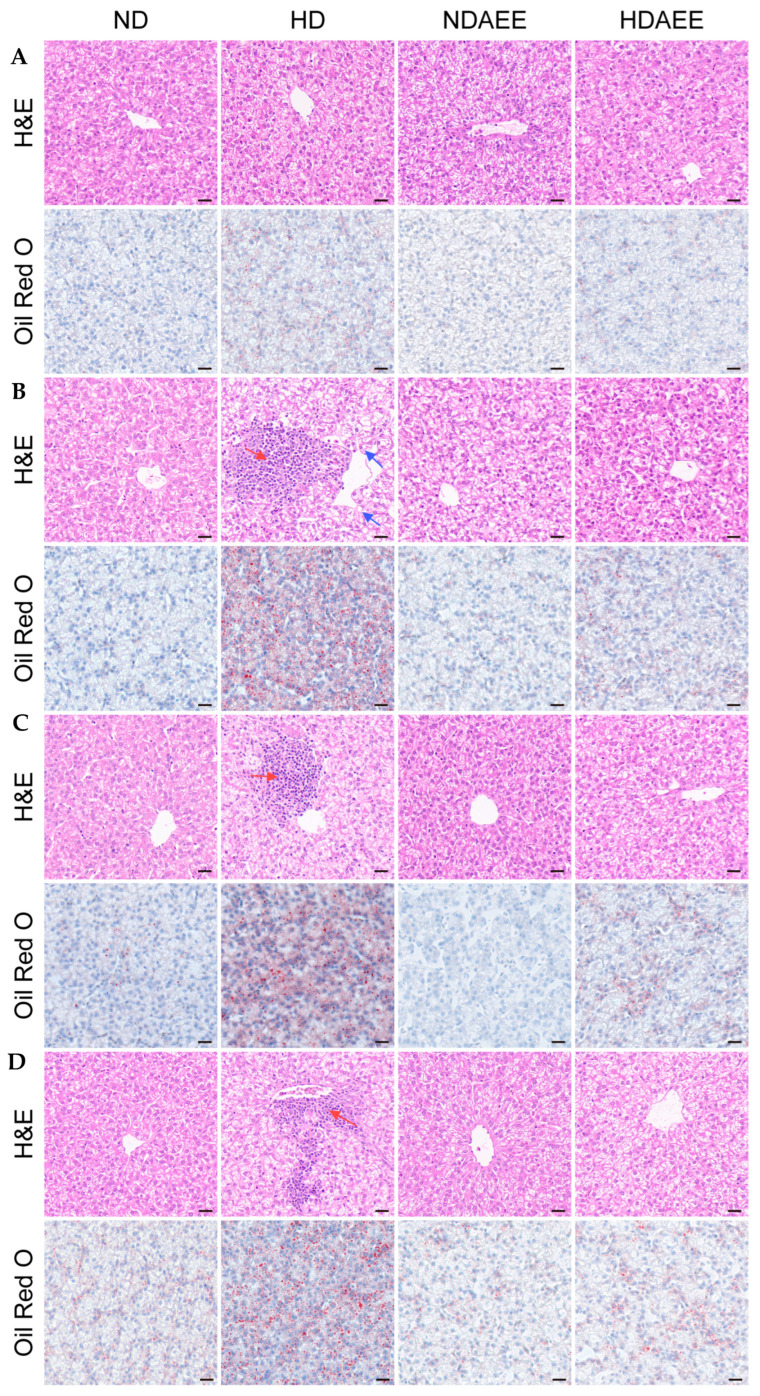
Effect of AEE on liver pathology and lipid accumulation in broilers under HSD stress as shown by H&E and Oil Red O staining of liver sections at 21, 28, 35, and 42 days of age (**A**–**D**). In H&E staining, pink represents the cytoplasm and blue the nucleus; red arrows indicate inflammatory cells, and blue arrows point to lipid vacuoles. In Oil Red O staining, the nucleus appears blue, and lipid droplets are stained red (400× magnification). Scale bar: 20 μm. Representative histopathological section of the liver from each group (n = 4).

After 28 days, the liver sections of broilers in the HD group began to show significant inflammatory cell infiltration (red arrows) and a small number of fatty vacuoles (blue arrows) on H&E staining (Figure 2). Oil red O staining showed clear accumulation of lipid droplets. After 35 and 42 days, the HD group still showed significant inflammatory cell infiltration and fat accumulation. However, after the administration of AEE, the cellular structure of liver tissue essentially returned to normal, with a significant reduction in inflammatory cell infiltration and fat deposition in the liver.

### 2.5. Identification of Differentially Expressed Metabolites

**Figure 3:**
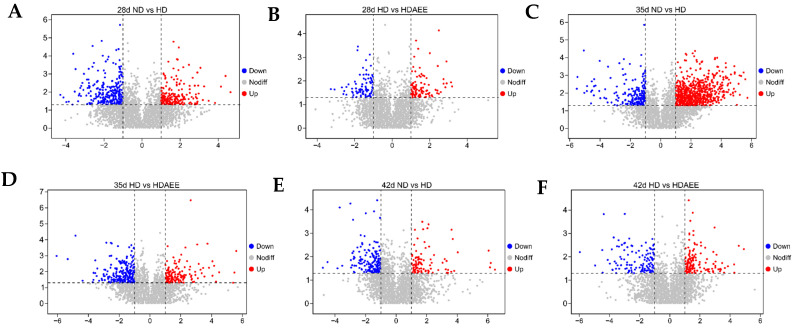
(**A**−**F**) Volcano plot screening for differentially expressed metabolites in the ND vs. HD and the HD vs. HD-AEE groups at 28, 35, and 42 days of age. Those with FC > 2 and *p* < 0.05 are marked in red, while those with FC < 0.5 and *p* < 0.05 are shown in blue. Differential metabolites of non-significance are marked in gray. For each group, n = 4.

**Figure 4:**
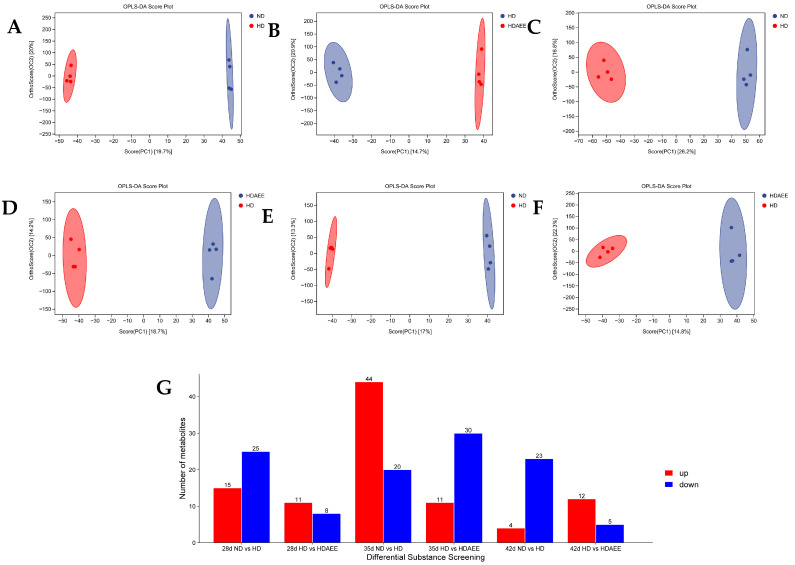
Analysis of significantly different metabolites in the ND vs. HD and HD vs. HD-AEE groups. (**A**,**B**) Metabolite profile scores from orthogonal partial least squares discriminant analysis (OPLS-DA) for each group at 28 days of age. (**C**,**D**) OPLS-DA for each group at 35 days of age. (**E**,**F**) OPLS-DA for each group at 42 days of age. PC1 is principal component 1, PC2 is principal component 2, and different colored points and ellipses represent samples and confidence intervals for different groupings. (**G**) Histogram of differential metabolites (n = 4).

To investigate the molecular mechanisms underlying the physiological effects of AEE under HSD conditions, we used an untargeted metabolomics approach to identify and compare the metabolites in liver samples from the ND, HD, and HD-AEE groups at 28, 35, and 42 days of age (Figure 3). Based on univariate statistical analyses, differential analysis of all detected metabolites (including unidentified metabolites) was performed by MS/MS in both positive- and negative-ion modes. Metabolites were visualized as volcano plots, with significantly up-regulated metabolites (FC > 2, *p* < 0.05) shown in red and significantly down-regulated metabolites (FC < 0.5, *p* < 0.05) represented in blue. Orthogonal projections to latent structures discriminant analysis (OPLS-DA) is a supervised model that can better assess the differences between two groups, improving the effectiveness and resolution of the model. A clear separation between groups was observed in the OPLS-DA score plots (Figure 4A–F), indicating good discrimination and within-group correlation among the liver samples. The total number of differential metabolites in each comparison group at 28, 35, and 42 days was determined by the criteria of OPLS-DA VIP > 1 and *p* < 0.05 (Figure 4G). At these days, 40, 64, and 27 metabolites were distinguished between the ND and HD groups, while 19, 41, and 17 metabolites differed between the HD and HD-AEE groups. Figure S1 shows the heatmap of cluster analysis for different metabolites in samples of different age groups, clearly showing the relative abundance of metabolites between groups.

### 2.6. Pathway Analysis of Differential Metabolites

**Figure 5:**
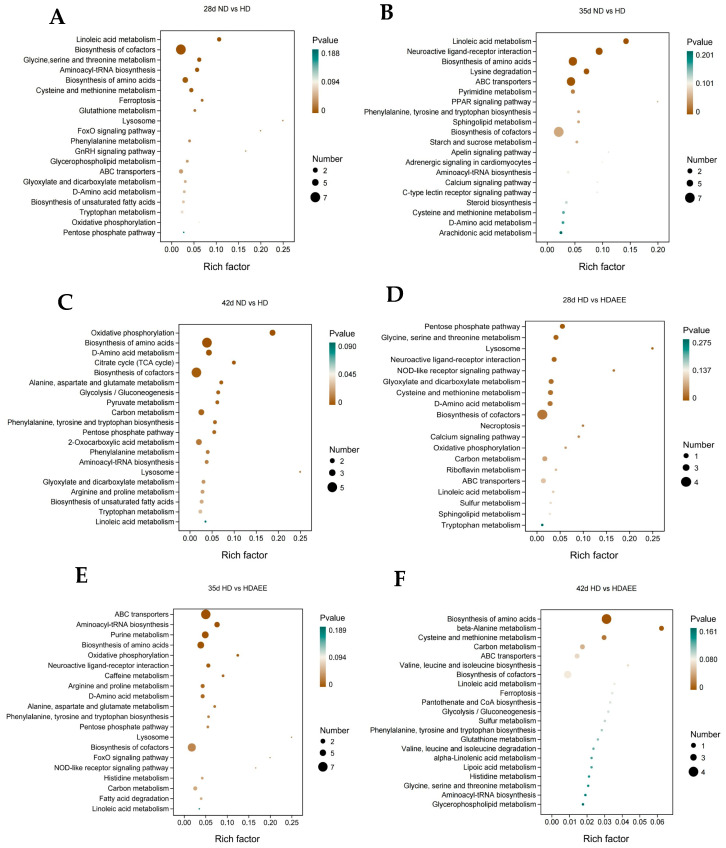
Comparative analysis of differential metabolic pathways in the liver of broilers from different treatment groups. (**A**–**C**) Factor diagram of KEGG enrichment analysis for the comparison of ND vs. HD at 28, 35, and 42 days of age. (**D**–**F**) Factor diagram of KEGG enrichment analysis for the comparison of HD vs. HDAEE at 28, 35, and 42 days of age (n = 4). The degree of enrichment of each pathway was determined by *p*-value and the number of metabolites, with significance assessed using the hypergeometric test (*p* < 0.05). The diameter of the circle indicates the number of metabolites. The varying colors, ranging from brown to green, symbolize the magnitude of the *p*-value. A lower *p*-value means a higher degree of significance of the enrichment level.

After 28, 35, and 42 days, a KEGG enrichment analysis of the differential metabolites was performed in the livers of broilers of the ND and HD groups (Figure 5A–C). Linoleic acid metabolism, amino acid biosynthesis, glutathione metabolism, oxidative phosphorylation, and glycine, serine, and threonine metabolism were highlighted as critical metabolic pathways after 28 days. Likewise, linoleic acid metabolism, arachidonic acid metabolism, amino acid biosynthesis, lysine degradation, and phenylalanine, tyrosine, and tryptophan biosynthesis were highlighted as crucial metabolic pathways after 35 days. Lastly, oxidative phosphorylation, amino acid biosynthesis, linoleic acid metabolism, and pentose phosphate pathway were highlighted as crucial pathways after 42 days. KEGG enrichment analysis of the differential metabolites in liver samples from the HD vs. HD-AEE groups, highlighted the pentose phosphate pathway, cysteine and methionine metabolism, oxidative phosphorylation, and linoleic acid metabolism as crucial pathways after 28 days (Figure 5D–F). After 35 days, the major metabolic pathways included oxidative phosphorylation, pentose phosphate, amino acid biosynthesis, fatty acid catabolism, and linoleic acid metabolism. After 42 days, the primary metabolic pathways included amino acid biosynthesis, cysteine and methionine metabolism, carbon metabolism, and linoleic acid metabolism. The characteristic types of differential metabolites in the various metabolic pathways are listed in Table 3 and Table 4.

## 3. Discussion

High stocking density (HSD), as a multifaceted stress factor, significantly affects the health and productivity of broilers and represents a major challenge for the development of the poultry industry [22,23]. Currently, there is a lack of effective management strategies for HSD stress in the market. Given this situation, AEE has attracted the attention of researchers due to its anti-inflammatory activity, antioxidant effectiveness and ability to positively regulate metabolism. After a comprehensive evaluation of its pharmacodynamics, pharmacokinetics, and toxicology, AEE was confirmed to be safe for widespread use in poultry production [24,25,26]. Since there are no successful studies on its potential hepatoprotective effects against HSD-induced liver injury in broilers, this study aims to evaluate and elucidate the effects of AEE on oxidative damage, inflammation, and energy metabolism in the livers of broiler chickens under HSD stress.

HSD can reduce feed intake, slow the growth rate, and thus reduce the production performance of broilers [27,28]. This study showed that the negative effects of HSD on broiler growth increased with age, especially during the period of 29 to 42 days of age, which is characterized by reduced ADFI and ADG, and increased FCR, which is consistent with previous findings [29]. During the rapid growth phase of broilers, there is a significant increase in their energy requirements, signaling the onset of HSD stress conditions [30,31]. Importantly, the addition of AEE effectively mitigated these adverse effects, especially from days 29 to 42, which is the critical growth period for broilers. This research highlights the potential of AEE as a feed additive to combat HSD stress, maintain optimal broiler growth performance, and promote animal welfare. It corroborates previous studies and confirms the pharmacological potential of AEE for addressing HSD challenges [20].

As the primary organ responsible for detoxification and nutrient metabolism in the body, the liver is particularly vulnerable to oxidative stress and inflammation caused by endogenous toxins and metabolic byproducts [32]. Under HSD conditions, the natural balance between ROS and the antioxidant system in the livers of broilers can be disrupted, leading to increased oxidative stress and damage [6,7]. GSH-Px, CAT, SOD, and T-AOC are commonly used indicators to assess oxidative damage and the ability to eliminate excess ROS from the body [33]. MDA is a compound formed when ROS attack unsaturated lipids in the liver, and its elevated levels are typically indicative of oxidative stress and redox imbalance [34]. In this study, the levels of antioxidant indicators SOD, GSH-Px, CAT, and T-AOC were significantly reduced in broilers under HSD conditions, but dietary AEE supplementation significantly increased these indicators. At the same time, the MDA level in HD broilers under HSD was significantly higher than in the ND group, while the MDA level in the HD-AEE group was significantly lower. Overall, AEE increased the free radical scavenging ability of the antioxidant enzyme system and alleviated the redox imbalance caused by HSD.

Oxidative stress and inflammation are closely related in many diseases, as they appear to occur simultaneously and promote each other at the site of injury [35]. ROS are involved in the signaling cascades during inflammation and can promote the expression of inflammatory mediators and enzymes by activating nuclear factor kappaB (NF-κB), including *COX-2*, *TNF-α*, *IL-6*, and *IL-1β* [10,11]. *COX-2* converts arachidonic acid into the inflammatory mediator PGE2, and *mPGES-1* plays a critical terminal rate-limiting role in its synthesis [36,37]. In this study, the HD group showed increased gene expression of *COX-2*, *mPGES-1*, *TNF-α*, *IL-1β*, and *IL-6* in liver compared to the ND group. However, AEE supplementation significantly reduced the expression of these inflammatory factors. Gene expression dynamics were most pronounced during the 35-day rapid growth period of broilers, with levels varying across time points, suggesting that environmental factors may modulate responses to changes in population density [38]. *IL-10* is an important anti-inflammatory cytokine that can limit the activation of innate immune cells and the production of cytokines, thereby reducing excessive, uncontrolled inflammatory responses [39]. Our study found that compared with ND, the expression of *IL-10* mRNA in the HD group was higher on days 21 and 28 and decreased on days 42; however, under HSD conditions, AEE increased *IL-10* mRNA after 42 days. This suggests that the initial increase in *IL-10* during early inflammation acts as a compensatory self-protective mechanism to curb excessive immune activation and mitigate inflammatory tissue injury. Subsequently, *IL-10* levels decline when the inflammatory response exceeds regulatory capabilities or microenvironmental changes impede *IL-10* synthesis [40]. However, AEE can increase *IL-10* expression and inhibit inflammatory responses. HSD can affect the synthesis, degradation, and transport of lipids in poultry and lead to disorders of lipid metabolism [41]. Excessive lipid accumulation has been proven to cause hepatotoxicity, which in turn leads to impaired liver function and worsens inflammatory reactions [42]. Studies have found that AEE can reduce blood lipid levels and fat accumulation in the liver by restoring impaired metabolic profiles and gut microbiota homeostasis [16]. Our results showed that HSD stress increased the relative lipid droplet area and inflammatory cell infiltration in the liver, which was reversed by the addition of AEE to the diet. Altogether, these results support the hypothesis that HSD stress causes liver inflammation and damage and mediates lipid metabolism disorder, while adding AEE to the diet effectively alleviates the negative effects of HSD in broilers.

To further investigate the influence of AEE on liver metabolism in broilers under HSD stress, we analyzed the liver metabolome of the HD, ND, and HD-AEE groups at three developmental time points. The results showed significant differences in linoleic acid, fatty acids, amino acids, pentose phosphate, and oxidative phosphorylation pathways related to inflammation, oxidative damage, and energy metabolism. Although the specific times at which these changes occurred varied, they were present across multiple time points.

Comparing KEGG pathway data at the three times, we found significant changes in amino acid metabolism between ND and HD groups. Amino acids actively function as signaling molecules to regulate metabolism, are involved in crucial biological processes such as glycolysis and the tricarboxylic acid cycle, and serve as essential substrates for the synthesis of proteins, nucleic acids and lipids [43,44]. Under HSD conditions, significant changes in amino acid metabolism were observed in broilers, characterized by accelerated protein degradation and slowed protein synthesis. In particular, L-tryptophan was significantly downregulated on days 28 and 42; L-proline on days 28, 35, and 42; and L-serine on day 28. The tryptophan derivative 5-hydroxytryptophan (5-HT) has been shown to maintain membrane fluidity in broilers exposed to oxidative stress [45]. Tryptophan supplementation improved FCR in poultry maintained at high stocking densities and reduced liver damage caused by oxidative stress [46]. Serine is a substrate for the synthesis of nucleotides, nicotinamide adenine dinucleotide phosphate (NADPH) and glutathione (GSH) [47]. As the primary non-protein thiol antioxidant in cells, GSH plays a crucial role in directly neutralizing free radicals and peroxides [48]. NADPH, generated via the pentose phosphate pathway (PPP), serves as a fundamental reducing agent for the glutathione cycle to maintain the antioxidant capacity of cells [49]. Recent studies have shown that L-proline can protect cells from oxidative stress in vivo and in vitro by regulating GSH-related redox homeostasis [50]. The downregulation of these factors further confirms that the birds were in a HSD-induced stress state from 28 days of age, and their antioxidant capacity was impaired. After 35 days, the increase in adrenaline levels in broilers, resulting from the conversion of L-phenylalanine to dopamine to meet increased energy demands via norepinephrine synthesis, coincided with upregulated levels of phosphoenolpyruvate (PEP), indicating enhancement of the glycolytic pathway as a mechanism for rapid ATP generation [51]. At the same time, increased activity of the lysine degradation pathway produced fumarate, NADPH, and acetyl coenzyme A (acetyl-CoA). Acetyl-CoA participates in the citrate cycle (TCA cycle), which generates ATP but also involves oxidative reactions that serve as part of the strategy for adaptive reprogramming of amino acid metabolism in cells to obtain energy under conditions of nutritional restriction [52].

The healthy growth and development of broilers also depends to a large extent on an adequate supply of polyunsaturated fatty acids (PUFAs), which must be fully covered by daily feed intake in order to meet their nutritional requirements [53]. Providing broilers with feeds rich in unsaturated fatty acids can effectively increase their antioxidant capacity, mitigate inflammatory responses, and optimize lipid metabolism, which contributes to better health and higher productivity [54,55]. In this study, we showed that HSD disrupted linoleic acid metabolism in the liver of broilers. Specifically, after 28 days, arachidonic acid, 10E,12Z-octadecadienoic acid, and 13(S)-hydroperoxyoctadecadienoic acid (13(S)-HPODE) were significantly downregulated; after 35 days, γ-linolenic acid was significantly downregulated; and after 42 days, linoleic acid was significantly downregulated. In addition, PGE2, LTB4, and 9-oxo-octadecadienoic acid (9-OxoODE) were significantly upregulated after 35 days. Compared to saturated fatty acids, PUFAs, (especially derivatives of linolenic acid) have an increased autoxidation rate, which allows them to quickly participate in the cellular energy production process [56]. This involvement is characterized by an accelerated conversion of linoleic acid into the derivatives of arachidonic acid during periods of stress, thereby increasing synthesis of the most important pro-inflammatory mediators PGE2 and LTB4. It is noteworthy that the concentration of 9-oxo-ODE in broilers from the HD group increased significantly in parallel with the MDA values, which is further evidence of an escalation of inflammation-related linoleic acid oxidation products and increased oxidative stress under HSD conditions. These results highlight the physiological mechanisms by which organisms cope with extreme environmental challenges by accelerating specific lipid metabolic pathways and increasing inflammatory responses [57]. However, linoleic acid provides a way for the liver to transport excess lipids to other tissues for storage or use; therefore, a deficiency in linoleic acid can lead to a reduction in lipoprotein synthesis and storage of fat in the liver [58,59]. Excessive lipid accumulation can impair the morphological transformation of mitochondria, thereby hindering the normal metabolic pathways of fatty acid oxidation and reducing ATP production [60]. This further confirms that disruptions in linoleic acid metabolism, increased inflammatory responses, and disruptions in normal lipid metabolic pathways can cause abnormal fat accumulation in the liver.

Intracellular ATP is synthesized primarily by oxidative phosphorylation in mitochondria, which occurs via the electron transport chain on the inner mitochondrial membrane [61]. Excessive production of ROS and abnormal accumulation of lipids can damage mitochondrial function, inhibit fatty acid β-oxidation and oxidative phosphorylation, reduce ATP production, and cause energy metabolism disorders [62]. Our results at 28 days suggest that the decline in adenosine diphosphate (ADP) could be an adaptive mechanism to increase ATP production to meet the increased energy demand. After 42 days, there was a significant decrease in ATP, pyrophosphate (PPi), and succinate in the oxidative phosphorylation pathway of the HD group. It is noteworthy that the simultaneous reduction in the intermediates, oxaloacetate and succinate, in the TCA cycle implies a deficiency of acetyl-CoA and an urgent increase in energy requirements. This change leads to a reduction in the production of nicotine adenine dinucleotide (NADH) and flavin adenine dinucleotide (FADH_2_), thereby affecting the function of the electron transport chain [63]. In addition, ROS reaction with components in the electron transport chain can inhibit electron transfer, slowing oxidative phosphorylation and significantly reducing ATP synthesis [64]. The HD-AEE group significantly increased the activity of the pentose phosphate pathway on days 28 and 35 compared with the HD group without treatment, which supported NADPH production and increased resistance to antioxidant stress [65]. Furthermore, by activating cysteine metabolism and the methionine cycle, the direct conversion ensured a sufficient supply of cysteine for GSH synthesis [66]. Significant increases in L-serine on day 28, L-proline on day 35 and 42, and glutathione on day 42 were observed in the HD-AEE group, which directly and strongly reflected an improvement in T-AOC, validating the positive effects of AEE in improving antioxidant defense mechanisms in broilers.

AEE supplementation significantly increased the levels of lipid metabolites, including γ-linolenic acid (28 days), 10E,12Z-octadecenoic acid (35 days), and phosphatidylcholine (PC) (42 days), while after 35 days, the levels of 1-hexadecanol and palmitoyl-L-carnitine decreased. This demonstrates that AEE improves linoleic acid metabolism, increases mitochondrial transport of fatty acids, promotes more efficient utilization of saturated fatty acids, reduces the risk of lipid peroxidation, and thus promotes energy production [67,68,69]. The HD-AEE group showed a dynamic optimization of energy metabolism with a significant increase in ATP content after 28 days and a sustained upregulation of ATP after 35 days. The observed increase in ATP content, coupled with reduced pyrophosphate and adenosine levels, suggests that AEE may optimize cellular energy metabolism, reduce the accumulation of pyrophosphate and adenosine, and thereby enhance ATP synthesis and utilization efficiency [70]. After 42 days, significant increases in the intermediates of carbon metabolism, glycerone-1,3-diphosphate and fructose-1,6-diphosphate, provided direct evidence of accelerated glycolysis and high metabolic efficiency, resulting in a rapid cellular response and increase in the ATP supply. When energy requirements are high, synergy between glycolysis and metabolic pathways, such as the PPP, is ensured and promoted [71]. Overall, AEE improves linoleic acid metabolism, effectively suppresses inflammation, accelerates the metabolism and consumption of fatty acids and their derivatives, improves the oxidation efficiency of fatty acids in mitochondria, and thereby promotes efficient ATP synthesis.

## 4. Materials and Methods

### 4.1. Animals and Experimental Design

A total of 360 healthy, weight-matched, one-day-old male broilers of the Arbor Acres (AA) breed were purchased from a commercial hatchery in Luoyang, China. The research was carried out at the Animal Research Unit of Henan University of Science and Technology. The broilers were randomly assigned to four experimental groups: normal density (ND, 14 broilers/m^2^), high density (HD, 22 broilers/m^2^), normal density with AEE supplementation (ND-AEE, 14 broilers/m^2^), and high density with AEE supplementation (HD-AEE, 22 broilers/m^2^). There were 10 replicates per treatment, with 7 broilers per replicate in the ND and ND-AEE groups, and 11 broilers per replicate in the HD and HD-AEE groups. The ND and HD groups received a basal diet, while the ND-AEE and HD-AEE groups received the same basal diet supplemented with 0.01% AEE. AEE was included in the treatment starting from the beginning of the experiment and continued until its conclusion. The 42-day experiment allowed animals free access to food and water and initially set the ambient temperature to 33 °C ± 2 °C, which was then reduced by 2 °C each week until it reached 24 °C ± 2 °C, where it was maintained. Relative humidity was kept between 40% and 60% with lighting available for 23 h daily and turned off from 7:00 to 8:00 each night. AEE (99.5% purity) was provided by Lanzhou Institute of Husbandry and Pharmaceutical Sciences, Chinese Academy of Agricultural Sciences, and the choice of AEE concentration of 0.01% was based on the finding that this concentration provided optimal antioxidant activity (Figure S2). The experimental diet for broilers was divided into two phases: days 1–21 and days 22–42. The formulated feed (Table 5) met all nutritional requirements. Immunization was carried out according to the standard broiler program, including regular disinfection, proper ventilation, and health monitoring.

### 4.2. Growth Performance

On days 14, 28, and 42, body weight and feed intake were recorded after an 8 h fasting period. ADG (average daily gain), ADFI (average daily feed intake), and FCR (feed conversion ratio) were calculated. Mortality rates were recorded daily and performance parameters adjusted accordingly.

### 4.3. Sample Collection

On days 21, 28, 35, and 42 of the experiment, 24 broilers (6 per group, with body weights close to average) were randomly selected from each of the four treatment groups. The birds were humanely euthanized by cervical dislocation for sampling and their livers were quickly removed. Liver samples were collected from the left lobe of the liver, placed in enzyme-free tubes, frozen in liquid nitrogen, and stored at −80 °C for further analysis.

### 4.4. Examination of Liver Histology

Hematoxylin and eosin (H&E) and Oil Red O staining were performed according to previously described methods [72,73]. The detailed procedures were as follows: Liver tissue blocks were collected from the same anatomical site and fixed immediately in 4% paraformaldehyde for a minimum of 24 h. After fixation, tissue samples were processed through a series of ethanol gradient dehydration, xylene clearing, and paraffin embedding to prepare 4 μm thick paraffin sections for H&E staining. For Oil Red O staining, frozen sections were prepared by embedding tissues in OCT compound and sectioning. The sections were then immersed in Oil Red O working solution for 8–10 min, followed by differentiation in 60% isopropanol and thorough rinsing with distilled water. After counterstaining with hematoxylin and additional rinsing with distilled water, the slides were mounted with glycerin gelatin. The stained tissue sections were observed under a microscope at 400× magnification, and images were captured using CaseViewer software (version 2.0).

### 4.5. Detection of Oxidative Damage Indicators

Oxidative damage indicators in liver samples were measured using test kits from Nanjing Jiancheng Bioengineering Institute (Nanjing, China) according to the manufacturer’s instructions. The activities of GSH-Px (A005-1-2), SOD (A001-3-2) and CAT (A007-1-1) in liver tissue were evaluated using colorimetric methods, WST-1 and ammonium molybdate methods, respectively. T-AOC in liver was determined using the ABTS method (A015-2-1), and the concentration of MDA (A003-1-2) was measured using the thiobarbituric acid (TBA) method.

### 4.6. Real-Time Quantitative PCR (RT-qPCR) Analysis of Liver mRNA Expression Levels

The relative abundance of *COX-2*, *mPGES-1*, *IL-1β*, *IL-6*, *TNF-α*, and *IL-10* mRNA in liver tissue was determined via RT-qPCR. Total RNA was isolated and purified with TRIzol reagent (Thermo Fisher Scientific, Ottawa, ON, Canada) and reverse transcribed into cDNA using the Evo M-MLV Mix Kit (Accurate Biology, AG11728, Changsha, China). RT-qPCR was conducted in 20 μL reaction volumes containing 2 μL cDNA template, 0.4 μL each of forward/reverse primers (Table 6), 10 μL 2X SYBR Green Pro Taq HS Premix (Accurate Biology, AG11701, Changsha, China), and 7.2 μL RNase-free water, on a CFX Connect Real-Time PCR system (Bio-Rad Laboratories, Hercules, CA, USA) with GAPDH as the reference gene. The reaction conditions were as follows: initial denaturation at 95 °C for 30 s, followed by 40 cycles of denaturation at 95 °C for 10 s and annealing at 60 °C for 30 s. The relative mRNA abundance of all genes was calculated using the 2^−ΔΔCT^ method [74].

### 4.7. Liver Metabolomics

Metabolites were extracted from liver samples using previously published methods [75]. Portions of metabolite extracts were pooled to create a quality control (QC) sample, which was used to correct biases in the results of pooled samples and compensate for instrumental errors caused by the analyzer. LC analysis was performed on a Vanquish UHPLC system (Thermo Fisher Scientific, Waltham, MA, USA) using an Acquity UPLC^®^ HSS T3 column (2.1 × 100 mm, 1.8 µm) (Waters, Milford, MA, USA). LC fractions were analyzed using a Thermo Orbitrap Exploris 120 mass spectrometer (Thermo Fisher Scientific, Waltham, MA, USA) in both positive and negative ionization modes [76]. The raw mass spectrometry files were converted to mzXML file format using the MSConvert tool of Proteowizard software (version 3.0.8789). A series of data processing steps including peak filtering and data standardization were performed and the observed spectra were compared to the metabolomic datasets to identify potential matches and annotate the peaks [77]. Lastly, the generated files were subjected to data analysis, including univariate and multidimensional statistical analyses, differential metabolite screening and correlation analysis, and KEGG pathway analysis.

### 4.8. Statistical Analyses

A one-way analysis of variance (ANOVA, SPSS 20.0, Chicago, IL, USA) was used for comparison and assessment of significance between different groups. The results are presented as mean ± standard error. *p* < 0.05 indicates a significant difference, and *p* < 0.01 indicates a very significant difference. The graphs were generated using GraphPad Prism 8.0 software (GraphPad Software Inc., San Diego, CA, USA).

## 5. Conclusions

**Figure 6:**
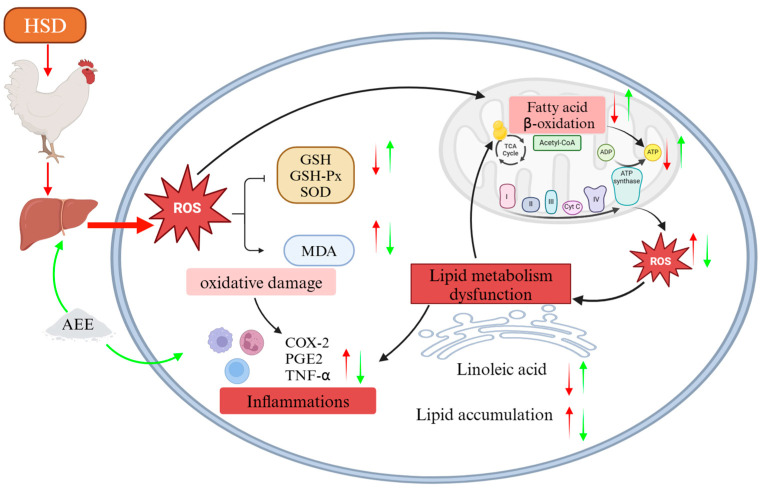
Diagram illustrating the proposed mechanism of how AEE alleviates oxidative damage, inflammation, and energy metabolism disorders in the livers of broilers under HSD stress. AEE restored normal liver morphology and mitochondrial function by upregulating the expression of key antioxidants, reducing inflammatory mediator expression, and inhibiting lipid peroxidation. This ensures normal lipid metabolism, decreases inflammatory responses and excessive lipid accumulation, and restores the energy metabolism balance in broilers under HSD stress. Red arrows indicate effects of HSD; green arrows represent effects of AEE. Upward and downward arrows denote increase and decrease, respectively.

In summary, our research results support the hypothesis that HSD stress leads to a decline in production performance of broilers through increased oxidative damage, inflammatory responses and energy metabolism disorders. AEE mitigates the HSD-mediated decline in broiler production performance by increasing the activities of SOD, GSH-Px, CAT, and T-AOC, promoting the expression of NADPH and GSH, reducing MDA levels, protecting the liver from oxidative damage, and maintaining the normal oxidative phosphorylation activity of mitochondria. In addition, AEE actively regulates the linoleic acid metabolic pathway, promotes the synthesis of γ-linolenic acid and PC, reduces the production of *COX-2* and *mPGES-1*, improves the efficiency of mitochondrial fatty acid oxidation, reducing excessive lipid accumulation in the liver, promotes ATP production, and effectively alleviates the metabolic imbalance caused by HSD (Figure 6). Our data suggest that AEE alleviates energy metabolism disorders caused by high stocking density by inhibiting oxidative damage and inflammation in the liver of broilers.

## Figures and Tables

**Table 1:** Effects of AEE on growth performance of broilers.

Items ^1^	ND	HD	ND-AEE	HD-AEE	SEM	*p*-Value
1–14 d						
ADFI, g	35.44	35.27	35.71	35.43	0.42	0.78
ADG, g	29.86	29.67	29.77	29.75	0.3	0.94
FCR	1.19	1.19	1.2	1.19	0.01	0.53
15–28 d						
ADFI, g	109.27 ^a^	105.04 ^b^	108.41 ^ab^	106.61 ^ab^	1.35	0.03
ADG, g	82.86	79.5	82.67	81.65	1.7	0.22
FCR	1.32	1.32	1.31	1.31	0.02	0.89
29–42 d						
ADFI, g	160.66 ^a^	145.9 ^b^	165.43 ^a^	155.63 ^a^	4.46	<0.01
ADG, g	94.63 ^a^	68.49 ^c^	95.12 ^a^	79.94 ^b^	4.37	<0.01
FCR	1.7 ^c^	2.13 ^a^	1.75 ^c^	1.95 ^b^	0.05	<0.01

^1^ ND, broilers at normal stocking density fed basal diet; HD, broilers at high stocking density fed basal diet; ND-AEE, normal-stocking-density group fed basal diet supplemented with 0.01% AEE; HD-AEE, high-stocking-density group fed basal diet supplemented with 0.01% AEE. ADG, average daily gain; ADFI, average daily feed intake; FCR, feed conversion ratio (feed: gain, g: g). Data are presented as mean ± SEM (n = 6), with different superscript letters (a, b, c) within the same row indicating statistically significant differences (*p* < 0.05).

**Table 2:** Effect of AEE on liver antioxidant function in broilers.

Items ^1^	ND	HD	ND-AEE	HD-AEE	SEM	*p*-Value
T-AOC (mmol/g)						
21 d	0.14	0.13	0.14	0.12	0.008	0.26
28 d	0.13 ^a^	0.11 ^b^	0.13 ^a^	0.13 ^a^	0.005	0.02
35 d	0.13 ^a^	0.11 ^b^	0.13 ^a^	0.13 ^a^	0.007	0.01
42 d	0.13 ^a^	0.11 ^b^	0.13 ^ab^	0.12 ^ab^	0.008	0.15
GSH-Px (U/mgprot)						
21 d	59.47	58.36	58.17	58.99	4.023	0.99
28 d	46.62 ^a^	36.63 ^b^	48.55 ^a^	47.65 ^a^	3.184	<0.01
35 d	62.03 ^a^	47.11 ^b^	58.13 ^a^	58.57 ^a^	3.78	0.01
42 d	66.43 ^a^	54.72 ^b^	65.1 ^a^	66.58 ^a^	3.693	0.01
CAT (U/mgprot)						
21 d	37.51	34.12	34.44	34.71	1.791	0.24
28 d	32.21	35.75	35.17	36.74	2.679	0.39
35 d	37.3 ^a^	31.5 ^b^	36.54 ^a^	36.88 ^a^	2.13	0.04
42 d	49.44 ^a^	37.25 ^b^	47.07 ^a^	46.2 ^a^	3.106	0.03
SOD (U/mgprot)						
21 d	31.3	30.68	30.33	29.62	1.538	0.75
28 d	27.37 ^ab^	24.99 ^c^	28.73 ^a^	26.61 ^b^	0.764	<0.01
35 d	27.2 ^a^	24.52 ^b^	29.09 ^a^	28.73 ^a^	1.011	<0.01
42 d	34.98 ^a^	32.78 ^ab^	34.36 ^ab^	32.64 ^b^	1.014	0.08
MDA (nmol/mgprot)						
21 d	0.33	0.36	0.35	0.35	0.034	0.82
28 d	0.31 ^b^	0.4 ^a^	0.34 ^b^	0.31 ^b^	0.026	<0.01
35 d	0.28 ^b^	0.35 ^a^	0.28 ^b^	0.3 ^ab^	0.029	0.07
42 d	0.44 ^b^	0.52 ^a^	0.4 ^b^	0.39 ^b^	0.027	<0.01

^1^ ND, broilers at normal stocking density fed basal diet; HD, broilers at high stocking density fed basal diet; ND-AEE, normal-stocking-density group fed basal diet supplemented with 0.01% AEE; HD-AEE, high-stocking-density group fed basal diet supplemented with 0.01% AEE. T-AOC: total antioxidant capacity; GSH-Px: glutathione peroxidase; CAT: catalase; SOD: superoxide dismutase; MDA: malondialdehyde. Data are presented as mean ± SEM (n = 6), with different superscript letters (a, b, c) within the same row indicating statistically significant differences (*p* < 0.05).

**Table 3:** Differentially expressed metabolites and related pathways in the liver of ND and HD chickens at 28, 35, and 42 days of age.

	Metabolite	*p*-Value	log2FC	Variation	Pathway
28 d ND vs. HD	Arachidonic acid	0.014	−0.659	Down	Linoleic acid metabolism
	10E,12Z-Octadecadienoic acid	0.032	−0.373	Down	Linoleic acid metabolism
	13(S)-HPODE	0.032	−1.311	Down	Linoleic acid metabolism
	L-Serine	0.001	−2.799	Down	Glycine, serine, and threonine metabolism
	L-Tryptophan	0.037	−0.419	Down	Glycine, serine, and threonine metabolism
	3-Phosphoglycerate	0.009	2.428	Up	Glycine, serine, and threonine metabolism
	L-Proline	0.012	−0.938	Down	Biosynthesis of amino acids
	Glutathione	0.013	−0.666	Down	Glutathione metabolism
	NADPH	0	−1.378	Down	Glutathione metabolism
	ADP	0.044	−0.92	Down	Oxidative phosphorylation
35 d ND vs. HD	γ-Linolenic acid	0.047	−0.336	Down	Linoleic acid metabolism
	10E,12Z-Octadecadienoic acid	0.038	−0.523	Down	Linoleic acid metabolism
	9-OxoODE	0.043	1.648	Up	Linoleic acid metabolism
	Prostaglandin E2	0.037	2.756	Up	Arachidonic acid metabolism
	Leukotriene B4	0.038	1.043	Up	Arachidonic acid metabolism
	L-Proline	0.022	−0.971	Down	Biosynthesis of amino acids
	(3S,5S)-3,5-Diaminohexanoate	0.038	0.671	Up	Lysine degradation
	N6-Acetyl-L-lysine	0.036	0.964	Up	Lysine degradation
	2-Keto-6-acetamidocaproate	0.034	1.722	Up	Lysine degradation
	Phosphoenolpyruvic acid	0.018	0.446	Up	Phenylalanine, tyrosine, and tryptophan biosynthesis
	L-Phenylalanine	0.046	0.726	Up	Phenylalanine, tyrosine, and tryptophan biosynthesis
	Epinephrine	0	3.736	Up	Neuroactive ligand–receptor interaction
42 d ND vs. HD	ATP	0.035	−2.251	Down	Oxidative phosphorylation
	Pyrophosphate	0.047	−0.206	Down	Oxidative phosphorylation
	Succinic acid	0.047	−1.668	Down	Oxidative phosphorylation
	Oxalacetic acid	0.024	−0.415	Down	Citrate cycle (TCA cycle)
	L-Tryptophan	0.024	−0.525	Down	Biosynthesis of amino acids
	L-Proline	0.008	−1.19	Down	Biosynthesis of amino acids
	Linoleic acid	0.035	−0.372	Down	Linoleic acid metabolism
	β-D-Fructose 6-phosphate	0.027	−1.381	Down	Pentose phosphate pathway
	2-Keto-D-gluconic acid	0.011	−0.146	Down	Pentose phosphate pathway

**Table 4:** Differentially expressed metabolites and related pathways in the liver of HD and HD-AEE chickens at 28, 35, and 42 days of age.

	Metabolite	*p*-Value	log2FC	Variation	Pathway
28 d HD vs. HDAEE	Glyceric acid	0.038	0.296	Up	Pentose phosphate pathway
	Ribose 1,5-bisphosphate	0.001	3.332	Up	Pentose phosphate pathway
	L-Serine	0.024	2.319	Up	Cysteine and methionine metabolism
	4-Methylthio-2-oxobutanoate	0.042	1.51	Up	Cysteine and methionine metabolism
	ATP	0.05	1.407	Up	Oxidative phosphorylation
	γ-Linolenic acid	0.047	0.133	Up	Linoleic acid metabolism
35 d HD vs. HDAEE	ATP	0.022	1.213	Up	Oxidative phosphorylation
	Pyrophosphate	0.031	−0.29	Down	Oxidative phosphorylation
	Adenosine	0.048	−0.924	Down	Neuroactive ligand–receptor interaction
	6-Phosphogluconic acid	0.022	1.101	Up	Pentose phosphate pathway
	2-Keto-D-gluconic acid	0	0.241	Up	Pentose phosphate pathway
	L-Proline	0.021	1.084	Up	Biosynthesis of amino acids
	Phosphoenolpyruvic acid	0.007	−0.56	Down	Biosynthesis of amino acids
	1-Hexadecanol	0.027	−0.528	Down	Fatty acid degradation
	Palmitoyl-L-carnitine	0.034	−0.821	Down	Fatty acid degradation
	10E,12Z-Octadecadienoic acid	0.016	0.762	Up	Linoleic acid metabolism
42 d HD vs. HDAEE	L-Proline	0.013	1.172	Up	Biosynthesis of amino acids
	Glyceric acid 1,3-biphosphate	0.004	1.425	Up	Carbon metabolism
	Fructose 1,6-bisphosphate	0.001	1.874	Up	Carbon metabolism
	Glutathione	0.043	0.336	Up	Cysteine and methionine metabolism
	Phosphatidylcholine	0.047	1.171	Up	Linoleic acid metabolism

**Table 5:** Formula and nutrient levels of the basal diets for the two growth periods.

Ingredient ^1^	Content (%)	
	1–21 d	22–42 d
Corn	52.79	57.78
Soybean meal	36.89	30
Zea gluten meal	0	2.43
Soybean oil	4	4
Sodium chloride	0.3	0.3
Choline chloride	0.3	0.26
Vitamin premix	0.03	0.03
Trace element premix	0.2	0.2
Stone powder	1.222	1.171
Dicalcium phosphate	1.912	1.623
DL-Methionine	0.265	0.106
L-Lysine	0.038	0.045
Wheat bran	2	2

^1^ The vitamin premix was provided on a per kg basis of the coordinated feed: VA 9500 IU, VD_3_ 62.5 μg, VE 30 IU, VK_3_ 2.65 mg, VB_1_ 2 mg, VB_6_ 6 mg, VB_12_ 0.025 mg, biotin 0.0325 mg, folic acid 1.25 mg, pantothenic acid 12 mg, and nicotinic acid 50 mg. The trace element premix was supplied on a per kg basis of the coordinated feed: copper 8 mg (CuSO_4_·5H_2_O), iron 80 mg (FeSO_4_), manganese 100 mg (MnSO_4_·H_2_O), selenium 0.15 mg (Na_2_SeO_3_), and iodine 0.35 mg (KI).

**Table 6:** Primer sequences.

Gene ^1^	Primer Sequence (5′–3′)	Length (nt)	GenBank Number
*COX-2*	F: CCGAATCGCAGCTGAATTCA R: GAAAGGCCATGTTCCAGCAT	116	NM_001277664.2
*mPGES-1*	F: AGGCTCAGGAAGAAGGCATT R: CACAGCTCCAAGGAAGAGGA	153	NM_001194983.1
*IL-1β*	F: ACTGGGCATCAAGGGCTA R: GGTAGAAGATGAAGCGGGTC	154	NM_214005.1
*IL-6*	F: GCTGCGCTTCTACACAGA R: TCCCGTTCTCATCCATCTTCTC	203	NM_204628.1
*TNF-α*	F: GAGCGTTGACTTGGCTGTC R: AAGCAACAACCAGCTA TGCAC	176	NM_214022.1
*IL-10*	F: AGAAATCCCTCCTCGCCAAT R: AAATAGCGAACGGCCCTCA	121	NM_001004414.2
*GAPDH*	F: TGCTGCCCAGAACATCATCC R: ACGGCAGGTCAGGTCAACAA	142	NM_204305.2

^1^ *COX-2* = cyclooxygenase-2; *mPGES-1* = microsomal prostaglandin E synthase-1; *TNF-α* = tumor necrosis factor-α; *IL-1β* = interleukin-1β; *IL-6* = interleukin-6; *IL-10* = interleukin-10; *GAPDH* = glyceraldehyde-3-phosphate dehydrogenase. F: forward primer; R: reverse primer.

## Data Availability

The raw data supporting the conclusions of this article will be made available by the authors on request.

## Supplementary Materials

The following supporting information can be downloaded at: https://www.mdpi.com/article/10.3390/ijms26051877/s1.
