# Supplementary material for: Aspirin Eugenol Ester Alleviates Energy Metabolism Disorders by Reducing Oxidative Damage and Inflammation in the Livers of Broilers Under High-Stocking-Density Stress
Source: Int J Mol Sci. 2025 Feb 21;26(5):1877. doi: 10.3390/ijms26051877 (PMC11899955; doi:10.3390/ijms26051877)
Supplement: Supplementary file 1 [file ijms-26-01877-s001.zip › ijms-3425386-supplementary.pdf]

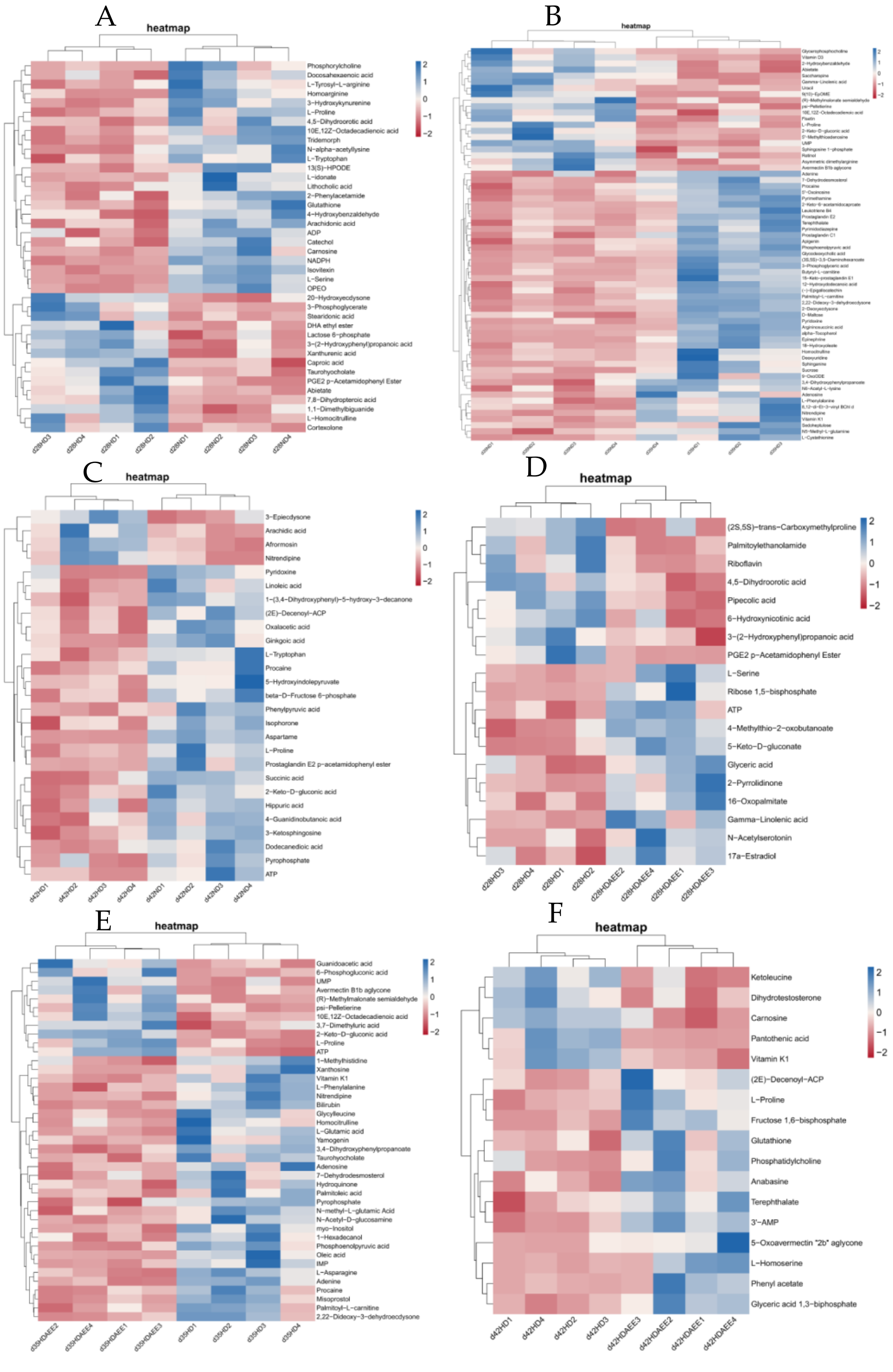

Figure S1. Heatmap and cluster analysis of differentially expressed metabolites in samples of different age groups. (**A-C**) ND, broilers at normal stocking density fed basal diet; HD, broilers at high stocking density fed basal diet; ND-AEE, normal stocking density group fed basal diet supplemented with 0.01% AEE; HD-AEE, high stocking density group fed basal diet supplemented with 0.01% AEE. Heatmaps of ND and HD chickens at 28, 35 and 42 days of age. (**D-F**) Heatmaps of HD and HD-AEE chickens at 28, 35 and 42 days of age (n = 4). Blue and red represent downregulated and upregulated genes, respectively. The darker the color, the greater the difference between the groups.

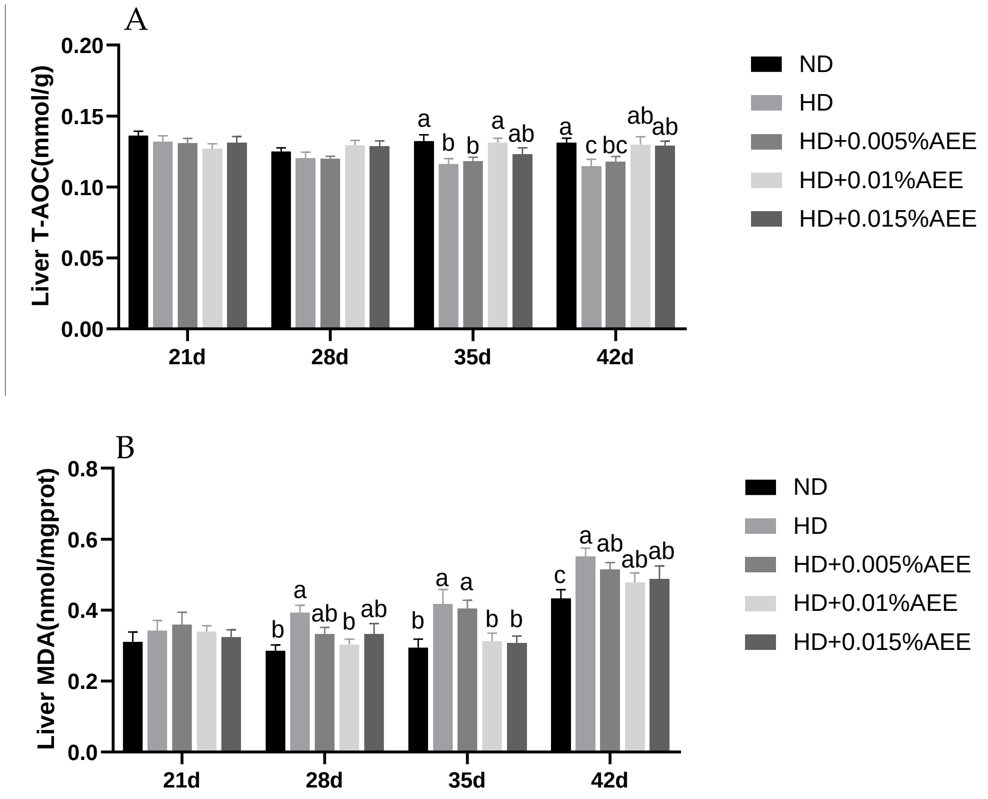

Figure S2. Effect of different concentrations of aspirin eugenol ester (AEE) added to the diet on the liver antioxidant capacity of high stocking density broilers. ND, broilers at normal stocking density fed basal diet; HD, broilers at high stocking density fed basal diet; HD-0.005% AEE, high stocking density group fed basal diet supplemented with 0.005% AEE; HD-0.01% AEE, high stocking density group fed basal diet supplemented with 0.01% AEE; HD-0.015% AEE, high stocking density group fed basal diet supplemented with 0.015% AEE. (**A**-**B**): T-AOC: total antioxidant capacity; MDA: malondialdehyde. Bars labeled with different letters (a, b, c) indicate significant differences across all groups ($P < 0.05$), with data presented as mean ± SEM (n = 6).
